# The evaluation of biological effects of artesunate on ovarian cancer using three-dimensional cell culture methods

**DOI:** 10.1007/s10561-026-10226-2

**Published:** 2026-08-20

**Authors:** Yijun Yuan, Xiaojing Luo, Longyu Tang, Tianwen He, Xinru Zou, Lu Feng, Bin Su, Jia Wu, Jun Li, Dongqin Xiao

**Affiliations:** 1 Department of Obstetrics and Gynecology, Research Institute of Tissue Engineering and Stem Cells, Beijing Anzhen Nanchong Hospital of Capital Medical University, Nanchong Central Hospital, The Second Clinical Medical College of North Sichuan Medical College, Nanchong, Sichuan People’s Republic of China 637000; 2 School of Medicine, University of Electronic Science and Technology of China, Chengdu, 610054 China

**Keywords:** Ovarian cancer, Artesunate, 3D cell culture, GelMA microspheres, Tumor microenvironment, Drug screening

## Abstract

Ovarian cancer (OC) is one of the most aggressive malignancies, with high mortality rates largely attributed to late-stage diagnosis, tumor heterogeneity, recurrence, and resistance to chemotherapy. Artesunate (ART), a water-soluble derivative traditionally used to treat malaria, has recently gained attention as a promising anticancer agent. Recently, studies have shown that ART can inhibit the migration and invasion of cancer cells. However, theses studies are always based on conventional two-dimensional (2D) culture models, which neglect the complex interactions between cells and the extracellular matrix (ECM) or in vivo microenvironment. To address these challenges, in this study, honeycomb-like gelatin methacryloyl (GelMA) hydrogel microspheres as a three-dimensional (3D) culture platform were introduced to mimic the native ECM microenvironment and assess the effects of ART on ovarian cancer cells. The results showed that these GelMA microspheres exhibited a uniform and highly porous structure, which supported cell adhesion, proliferation, and migration. Notably, 3D culture promoted DNA synthesis and induced G1-to-S phase transition, indicating enhanced proliferative capacity. Furthermore, ovarian cancer cells cultured in 3D exhibited stronger invasive and chemoresistant phenotypes. RNA sequencing revealed that after ART treatment, tumor-related proliferation and invasion genes of ovarian cancer cells were significantly up-regulated under 3D culture compared with 2D culture. This might be related to the up-regulation of TNF signaling pathway, NF-kappa B signaling pathway, and Pl3K-Akt signaling pathway. Correspondly, epithelial-mesenchymal transition (EMT) of ovarian cancer cells was markedly enhanced when cultured on GelMA hydrogel microspheres after ART treatment. These effects might be attributed to the altered microenvironment provided by the GelMA hydrogel microspheres, which could be responsible for the enhanced resistance of ovarian cancer cells to ART. These findings suggest that, compared to traditional 2D. These findings suggest that, compared to traditional 2D culture, the 3D extracellular matrix-based system better mimics the in vivo tumor architecture, thereby improving the sensitivity of anticancer drug screening and offering a more reliable platform for evaluating drug resistance and tumor cell heterogeneity.

**Supplementary Information:**

The online version contains supplementary material available at https://doi.org/10.1007/s10561-026-10226-2.

## Introduction

Ovarian cancer (OC) is one of the deadliest cancers affecting the female reproductive system worldwide. Its high mortality rate is primarily attributed to late-stage detection, inter- and intra-tumoral heterogeneity, high recurrence, and limited effective treatment options (Sung et al. 2021; Qian et al. 2023). Depending on the cell of origin, ovarian cancer can be classified into three types: epithelial ovarian cancer (EOC), germ cell ovarian cancer, and sex cord-stromal ovarian cancer. The vast majority of malignant ovarian cancers belong to the epithelial ovarian cancer subtype, accounting for more than 90% of all cases (Lheureux et al. 2019; Berney et al. 2020). Among these, the ES-2 ovarian cancer cells are of the clear cell carcinoma (OCCC) type, which is a specific histological subtype of epithelial ovarian cancer (EOC) (Gan et al. 2023 Jan). Approximately 70% of patients are diagnosed at an advanced stage (FIGO stage III and IV) with distant metastasis (Cortez et al. 2018). The majority of patients develop recurrent disease that is resistant to chemotherapy, resulting in a global 5-year survival rate of approximately 30–40%, and today the standard treatment for ovarian cancer is primarily surgery followed by platinum-based chemotherapy (Yang et al. 2020). Drug resistance is a great challenge in the treatment of ovarian cancer and a major cause of poor prognosis (Wang et al. 2024). Therefore, the search for new drugs or therapeutic strategies has become a major challenge in the clinical management of ovarian cancer.

Artesunate (ART), a water-soluble derivative of artemisinin produced via semi-synthetic processes, has been widely used as a first-line drug in clinical treatment of malaria (Sun et al. 2019). Recently, ART has been repurposed as a potential anticancer agent due to its ability to generate reactive oxygen species (ROS) by reacting with ferrous catalysts through its endoperoxide bridge, thereby inhibiting cancer cell proliferation (Zhong et al. 2022). Previous studies have demonstrated that ART synergizes with low-dose sorafenib to induce ferroptosis in hepatocellular carcinoma (HCC) both in vitro and in vivo (Li et al. 2021). Huang et al. confirmed that ART inhibits colorectal cancer cell proliferation by promoting excessive ROS production, leading to cellular senescence and autophagy (Huang et al. 2022). In ovarian cancer, ART has been shown to sensitize cells to cisplatin in vitro by inducing oxidative stress, DNA double-strand breaks, apoptosis, and ferroptosis (Zhao et al. 2020). Furthermore, ART suppresses RAD51 expression and the ATM/ATR-mediated DNA damage response, thereby impairing DNA repair mechanisms in ovarian cancer cells (Wang et al. 2015; Berdelle et al. 2011). Our previous study also identified the HOXC11/PROM2/PI3K/AKT axis as a novel regulatory mechanism of ART-induced ferroptosis in ovarian cancer (Li et al. 2024a).

However, most of the studies mentioned have been based on two-dimensional (2D) cell cultures. While 2D models are widely used in cancer research, their simplified tumor structures and microenvironments often alter biological characteristics like morphology, proliferation, and metabolism, limiting their ability to mimic the heterogeneity and complex cellular interactions of tumors in vivo (Fontoura et al. 2020). In contrast, three-dimensional (3D) cell cultures offer a more accurate simulation of the ECM microenvironment, enabling cells to form structures similar to those in vivo. 3D cultures better reflect tumor growth patterns, drug penetration, and resistance to treatments by replicating cell–cell and cell-ECM interactions (Kyriakopoulou et al. 2023). 2D cultures lack these interactions and spatial architecture, often leading to an overestimation of drug sensitivity, thus limiting their application in drug screening. 3D models, on the other hand, provide a better prediction of drug responses. For example, Yoshinori et al. reported that breast cancer cells in 3D cultures were resistant to chemotherapy drugs like paclitaxel and doxorubicin, while showing high sensitivity in 2D cultures (Imamura et al. 2015). 3D culture methods include both scaffold-free and scaffold-based systems. Scaffold-free cultures, such as hanging drop or low-adhesion plate methods, allow cells to self-assemble into spheroids, simulating cell aggregation and nutrient gradients (Ferreira et al. 2018; Yi et al. 2021; Sutherland 1988; Ekert et al. 2014). However, this approach faces challenges like uncontrolled spheroid size and structural instability. Scaffold-based 3D cultures, using biomimetic materials, provide better simulation of tumor tissue mechanical properties and matrix environment (Griffith and Swartz 2006; Caliari and Burdick 2016). Gelatin methacrylate (GelMA), a modified form of gelatin, is widely used as a scaffold material due to its biocompatibility, adjustable mechanical properties, and photopolymerization ability (Worthington et al. 2015; Banks et al. 2015; Lewis et al. 2017). Recently, GelMA-based hydrogel microspheres have gained attention as 3D culture platforms due to their controlled size, diverse structures (e.g., porous, hollow, core–shell), and ease of cell encapsulation (Zhang et al. 2022). These microspheres not only enhance cell attachment and nutrient exchange but also facilitate tissue reconstruction by forming porous structures. GelMA microspheres provide anchorage points that promote cell proliferation, migration, and spatial organization, and they gradually degrade in response to cell activity, reflecting good tissue adaptability (Yue et al. 2015; Liu et al. 2020). Thus, GelMA-based 3D culture systems offer a promising approach to modeling ECM microenvironment and support the development of new anticancer drugs.

Hydrogel microspheres have several advantages: (1) uniform structure; (2) strong compositional versatility, as they can be made from materials like gelatin, hyaluronic acid, and poly(lactic acid); (3) diverse drug loading methods, allowing for drug release through mixing or encapsulation in nanoparticles, followed by chemical conjugation to the microsphere surface; and (4) suitability for large-scale production, low cost, and high homogeneity (Wu et al. 2022). GelMA (gelatin methacryloyl) is a natural biomaterial commonly used in cancer research, particularly for 3D hydrogel scaffolds. Among them, gelatin, as a natural hydrophilic polymer, is formed by the partial hydrolysis of native collagen. It is widely found in the extracellular matrix (ECM) of most tissues and is one of the major components thereof (Young et al. 2005). Gelatin contains the Arg-Gly-Asp (RGD) peptide sequence, a sequence that promotes cell adhesion, spreading, and differentiation. Gelatin also contains a matrix metalloproteinase (MMP) recognition sequence, which allows it to be degraded by the appropriate enzymes, thus performing its function of dynamically regulating the extracellular matrix in organisms (Nazir et al. 2021; Bigi et al. 2001). GelMA (gelatin methacryloyl) is a natural biomaterial commonly used in cancer research, mainly for 3D hydrogel scaffolds. GelMA hydrogels are derived from collagen-containing gelatin, modified with methacryloyl, and then prepared by photo cross-linking of photoinitiators with an aqueous solution of GelMA prepolymers under UV irradiation or visible light to form a stable hydrogel network. This property makes it ideal for ECM microenvironment mimicking (TME) scaffolds. Its tunable mechanical and biochemical properties, biocompatibility, and ability to support cell growth make it highly valuable in cancer cell behavior studies, tumor invasion research, and drug screening (Abuwatfa et al. 2024). To better simulate the in vivo ECM microenvironment and promote ovarian cancer cell growth and invasiveness, porous GelMA microspheres were prepared for cell seeding. In porous GelMA microspheres, cells not only attach to the surface but also form more complex 3D structures within the microspheres, mimicking in vivo cell aggregation and migration. This 3D culture model more accurately reflects the biological behaviors of tumor cells, such as invasiveness, metastasis, and drug resistance. Additionally, the pore structure of the microspheres facilitates uniform nutrient distribution and waste removal, avoiding the problems of cell aggregation and oxygen deficiency common in 2D culture (Li et al. 2022).

In this study, microfluidic technology was employed to fabricate structurally uniform, porous, honeycomb-like GelMA hydrogel microspheres. This three-dimensional (3D) culture platform was then utilized to evaluate the effects of artesunate (ART) on ovarian cancer cells. Using this system, the anticancer efficacy of ART—particularly in terms of drug resistance and cellular invasiveness—was assessed with greater precision. The uniform architecture and tunable properties of the microspheres not only improved the cellular microenvironment but also enhanced the accuracy of drug screening. Our findings suggest that, compared to conventional two-dimensional (2D) cultures, the 3D honeycomb-like GelMA microsphere system promotes accelerated cell cycle progression, increased proliferation and migration, while maintaining cellular stemness, invasiveness, and drug resistance. Transcriptomic GSEA analysis of ovarian cancer cells cultured in 3D further revealed that tumor-promoting pathways were more significantly activated following ART treatment than in 2D cultures. Therefore, GelMA microspheres represent a promising 3D culture platform not only for evaluating the inhibitory effects of ART but also for advancing precision therapy and novel drug development for ovarian cancer.

## Results and discussion

### Preparation and characterization of porous GelMA microspheres

Microfluidic flow-focusing devices (FFD) are widely used to generate monodisperse droplets and microgels with controlled size, shape, and composition for various biomedical applications. They have advanced material science and provided integrated, miniaturized, and automated solutions for high-throughput 3D scaffolds (Jackson et al. 2017; Bian et al. 2021). Hydrogels produced using microfluidic technology can serve as 3D culture matrices for various cells, making them particularly suitable for the construction of in vitro tumor models (Xu et al. 2020). Inspired by the honeycomb structure, this study utilized microfluidic technology to fabricate GelMA microspheres. Oil droplets were generated as the continuous phase, while a 10% GelMA solution containing 3% lithium phenyl (2,4,6-trimethylbenzoyl) phosphate (LAP) as the photoinitiator served as the dispersed phase. Monodisperse droplets of identical size were produced by equal-speed batch processing and exposed to 405 nm UV light for photopolymerization in a synchronized crosslinking chamber. The droplets were then collected, washed, and lyophilized to obtain GelMA microspheres (Tan et al. 2023). Previous studies have demonstrated the suitability of GelMA hydrogels for simulating breast cancer metastasis characteristics in 3D in vitro systems, particularly in terms of invasiveness and chemotherapy response (Bock et al. 2023). The mechanical and morphological properties of the hydrogel were optimized by adjusting the GelMA ratio. Compression tests indicated that 10% GelMA hydrogel stiffness falls within the reported range for breast tissue, making it suitable for modeling the viscoelasticity of breast tissue. In both cell lines, cells cultured on 10% GelMA hydrogels showed higher proliferation rates compared to those on 15% GelMA, indicating the robustness of 10% GelMA as a long-term cell culture system (Hoarau-Véchot et al. 2016). Therefore, a 10% GelMA concentration was chosen for the subsequent preparation of GelMA microspheres in this study.

**Fig. 1:**
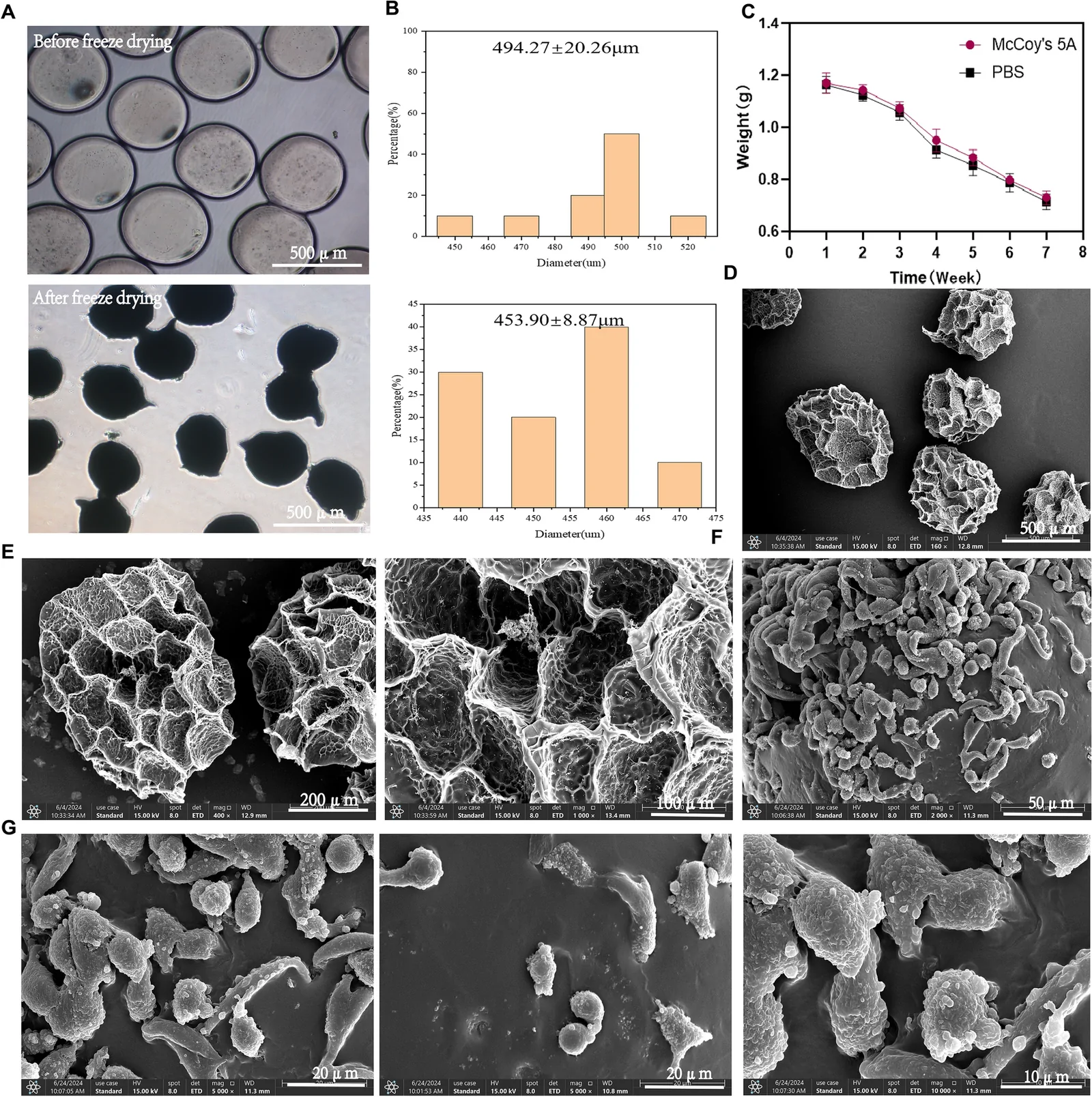
Structural and morphological characterization of porous GelMA microspheres. **A** General view of microspheres before and after freeze-drying. **B** Particle size distribution of microspheres before and after freeze-drying. **C** Degradation of GelMA microspheres in culture medium and PBS(n = 10). **D, E** SEM scan of microspheres after freeze-drying. **F, G** Ovarian cancer cells cultured in GelMA microspheres after dehydrated culture as viewed by SEM (Different magnifications)

After freeze-drying, the microspheres were observed using an inverted optical microscope. As shown in the images, GelMA microspheres displayed uniform size and consistent shape both before and after freeze-drying. After freeze-drying, the microspheres showed a noticeable reduction in diameter, from an average of 494.27 ± 20.26 μm to 453.90 ± 8.87 μm. However, when placed in physiological saline or PBS buffer, the microspheres largely restored their original size, demonstrating a reversible expansion property that is crucial for supporting cell adhesion on both the surface and within the microspheres (Fig. 1A, B). After soaking the microspheres in culture medium and PBS, their weights were measured at weekly intervals from week 1 to week 7. The results indicate that the microspheres remain relatively stable in both culture medium and PBS, degrade slowly, and are suitable for cell culture (Fig. 1C). Scanning electron microscopy (SEM) further revealed interconnected porous structures on both the surface and inside the GelMA microspheres, exhibiting a typical honeycomb morphology (Fig. 1 D, E). This loose and porous structure facilitates cell growth and migration, significantly enhancing the 3D biological functionality of the culture system. Additionally, the porosity of the microspheres aids in the exchange of nutrients and metabolic waste, providing a more stable microenvironment for cells (He et al. 2022). Upon cell seeding, time-lapse observations on day 5 showed that cells not only proliferated on the surface of the microspheres but also migrated into the internal pores, indicating that the porosity of the GelMA microspheres supports the 3D growth of cells and highlights the superiority and application potential of this 3D culture system (Fig. 1 F, G). Therefore, this loose and porous structure provides ample space for cell adhesion and proliferation, effectively enhancing intercellular interactions as well as interactions between cells and the extracellular matrix (ECM), thereby more accurately recapitulating the complexity of the ECM microenvironment under in vivo conditions. Sasaki et al. (2024) (Table 1).

**Table 1:** Primers for qRT-PCR assay

Primer name	Sequence (5 ‘-3’)
ANGPTL4-F	GTCCACCGACCTCCCGTTA
ANGPTL4-R	CCTCATGGTCTAGGTGCTTGT
ANXA8-F	ATGGCCTGGTGGAAATCCTG
ANXA8-R	TCATGCTGCTGAGGGTCTTG
EGR3-F	GACATCGGTCTGACCAACGAG
EGR3-R	GGCGAACTTTCCCAAGTAGGT
SEMA3B-F	ACATTGGTACTGAGTGCATGAAC
SEMA3B -R	GCCATCCTCTATCCTTCCTGG
PCDH7-F	CGGCCCGTGTATTGTCTAGC
PCDH7-R	TAGCCAGGGTGTCCAAATCT
VCAN-F	GTAACCCATGCGCTACATAAAGT
VCAN-R	GGCAAAGTAGGCATCGTTGAAA
E-cadherin-F	CGAGAGCTACACGTTCACGG
E-cadherin-R	GGGTGTCGAGGGAAAAATAGG
N-cadherin-F	AGCCAACCTTAACTGAGGAGT

N-cadherin-R	GGCAAGTTGATTGGAGGGATG
Bcl2-F	GGTGGGGTCATGTGTGTGG
Bcl2-R	CGGTTCAGGTACTCAGTCATCC
Bax-F	CCCGAGAGGTCTTTTTCCGAG
Bax-R	CCAGCCCATGATGGTTCTGAT
CD133-F	GGTAGTGTTGTACTGGGCCAAT
CD133-R	GGTAGTGTTGTACTGGGCCAAT
Ki67-F	ACGCCTGGTTACTATCAAAAGG
Ki67-R	CAGACCCATTTACTTGTGTTGGA

In summary, the honeycomb-structured porous GelMA microspheres, fabricated via microfluidic technology, exhibit excellent porosity, biocompatibility, and degradability, making them ideal for use as 3D cell culture matrices. This method allows for large-scale production while maintaining the original phenotype of tumor cells and simulating the physiological tumor microenvironment effectively. By preserving the interactions between cells and the extracellular matrix, these microspheres provide a reliable platform for studying tumorigenesis mechanisms and exhibit significant potential for anticancer drug screening. Thus, this technology offers an efficient and easily implementable solution for tumor biology research.

### Reconstruction of standard 3D tumor model and evaluation of ART in the 3D model

**Fig. 2:**
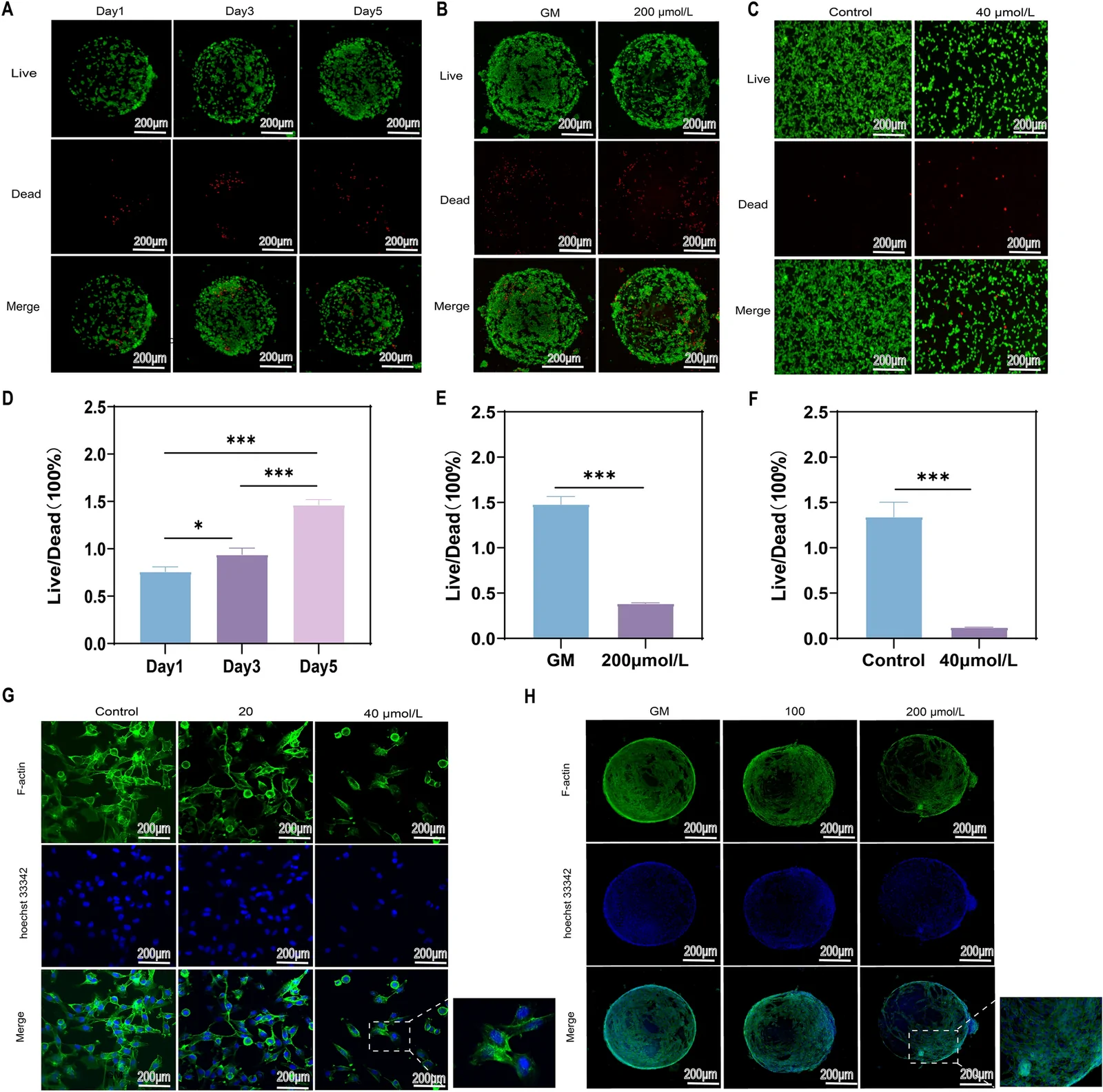
Biocompatibility evaluation of ART on ovarian cancer cells under GelMA microspheres and two-dimensional culture conditions. **A** Live-dead staining of ovarian cancer cells on days 1, 3, and 5 after implantation on the surface of GelMA microspheres. **B** Live-dead staining of ovarian cancer cells on GelMA microspheres before and after artesunate treatment. **C** Live-dead staining of two-dimensional cultured cells by artesunate. **D** Quantitative Analysis Results of Cell Viability Staining Using GelMA Microspheres. **E, F** Quantitative analysis results of live/dead staining following treatment of ovarian cancer cells with ART. **G** Staining of ovarian cancer cells cultured in two dimensions with phalloidin. **H** Staining of ovarian cancer cells on GelMA microspheres with phalloidin

In 2D cell cultures, cells grow only on the flat surface; whereas in 3D cultures, cells can migrate into the matrix and form contacts in all dimensions (Rafii et al. 2018). To evaluate the biocompatibility of GelMA microspheres with ovarian cancer cells, we seeded ovarian cancer cells onto the surface of the GelMA microspheres. After culturing the cells for 1, 3, and 5 days, we observed through live/dead staining that, despite the relatively long cell cycle, the cells were still able to adhere well to the microsphere surface and proliferate. By day 5 of culture, the microspheres were almost completely covered by cells (Fig. 2A, D), which displayed a plump morphology and tight arrangement, forming a quasi-three-dimensional encapsulating structure. This observation suggests that the cells possess a high proliferative density and efficient spatial utilization in this microenvironment. These results indicate that 3D GelMA microspheres provide an excellent matrix for ovarian cancer cells, supporting efficient 3D proliferation and growth, thereby demonstrating their potential for tumor model construction.To further evaluate the therapeutic effects of ART, Ovarian cancer cells were treated with different concentrations of artesunate respectively, “GM” refers to 3D GelMA microsphere culture, while “Control” refers to the blank group in 2D culture(Fig. 2B, C).The results showed that ART significantly induced cell death in both 2D and 3D cultures. However, under 3D culture conditions, higher drug concentrations(200 μmol/L) were required to achieve a similar level of cell death as observed in 2D cultures (40 μmol/L) (Fig. 2E,F). This suggests that, compared to two-dimensional culture, the interactions among cells and between cells and the extracellular matrix (ECM) are more complex in the 3D GelMA microspheres culture system. The enhanced ECM barrier may hinder drug diffusion and penetration, thereby reducing cellular sensitivity to artesunate and necessitating a higher drug concentration to achieve comparable therapeutic effects. This phenomenon implies that 3D GelMA microspheres systems may more accurately represent the drug resistance characteristics of in vivo tumors in drug screening. Mechanical signals are transduced through integrins on the cell surface and directed to the cytoskeletal proteins, generating prestress. The cell nucleus-cytoskeleton connection serves both to anchor the nucleus and to provide the main force for nuclear movement (Liu et al. 2016). Upon observing changes in cytoskeletal morphology using phalloidin staining, ovarian cancer cells cultured in 2D exhibited significantly increased cell death rates with increased ART concentrations, and the cell morphology underwent notable changes, transitioning from spindle-shaped to round, with more severe morphological degradation (Fig. 2G); In contrast,we found that ovarian cancer cells seeded onto the GelMA microspheres exhibited better spreading and a more pronounced layered proliferative state. After ART treatment, despite some cell death, the cell morphology remained relatively intact as the drug concentration increased (Fig. 2H). These results suggest that the 3D GelMA microsphere culture system, to some extent, can mitigate drug-induced cell morphological degradation, whereas 2D cultures are more susceptible to the effects of drug concentration, resulting in incomplete cell morphology and higher cell death.

### Evaluation of artesunate screening on GelMA microspheres and two-dimensionally cultured ovarian cancer cells

Maintaining the characteristics of tumor cells in their native biological state is crucial for constructing effective 3D tumor culture models. The stem-like properties of tumor cells, as the foundation of their malignant behavior and heterogeneity, directly influence tumor initiation, progression, and response to therapy (Braccini et al. 2022). Therefore, preserving the stem cell characteristics of tumor cells is not only essential for understanding tumor pathogenesis but also indispensable in drug screening and efficacy assessment. Previous studies have shown that cancer stem cell markers expressed in 3D cancer matrix models closely resemble in vivo conditions (He et al. 2022).To further investigate the effects of the GelMA microsphere culture system on the biological characteristics of ovarian cancer cells,we seeded ovarian cancer cells onto the surface of GelMA microspheres and cultured them under 3D conditions for 5 days. Subsequently, the microspheres were lysed, and the cells were collected. A portion of the collected cells was used for functional assays, including cell proliferation, colony formation, scratch assay, cell cycle analysis, apoptosis detection, and Transwell migration and invasion assays, To evaluate the efficacy of artesunate ester in drug screening using GelMA microspheres and ovarian cancer cells cultured in two dimensions, as well as changes in the biological behaviour of the ovarian cancer cells, including growth, migration and invasion. The remaining cells were used for RNA extraction to analyze the differential gene expression between 3 and 2D culture conditions, with the aim of elucidating the regulatory mechanisms of 3D culture on the biological characteristics and gene expression of ovarian cancer cells. This would provide experimental evidence for tumor pathogenesis and drug screening.

**Fig. 3:**
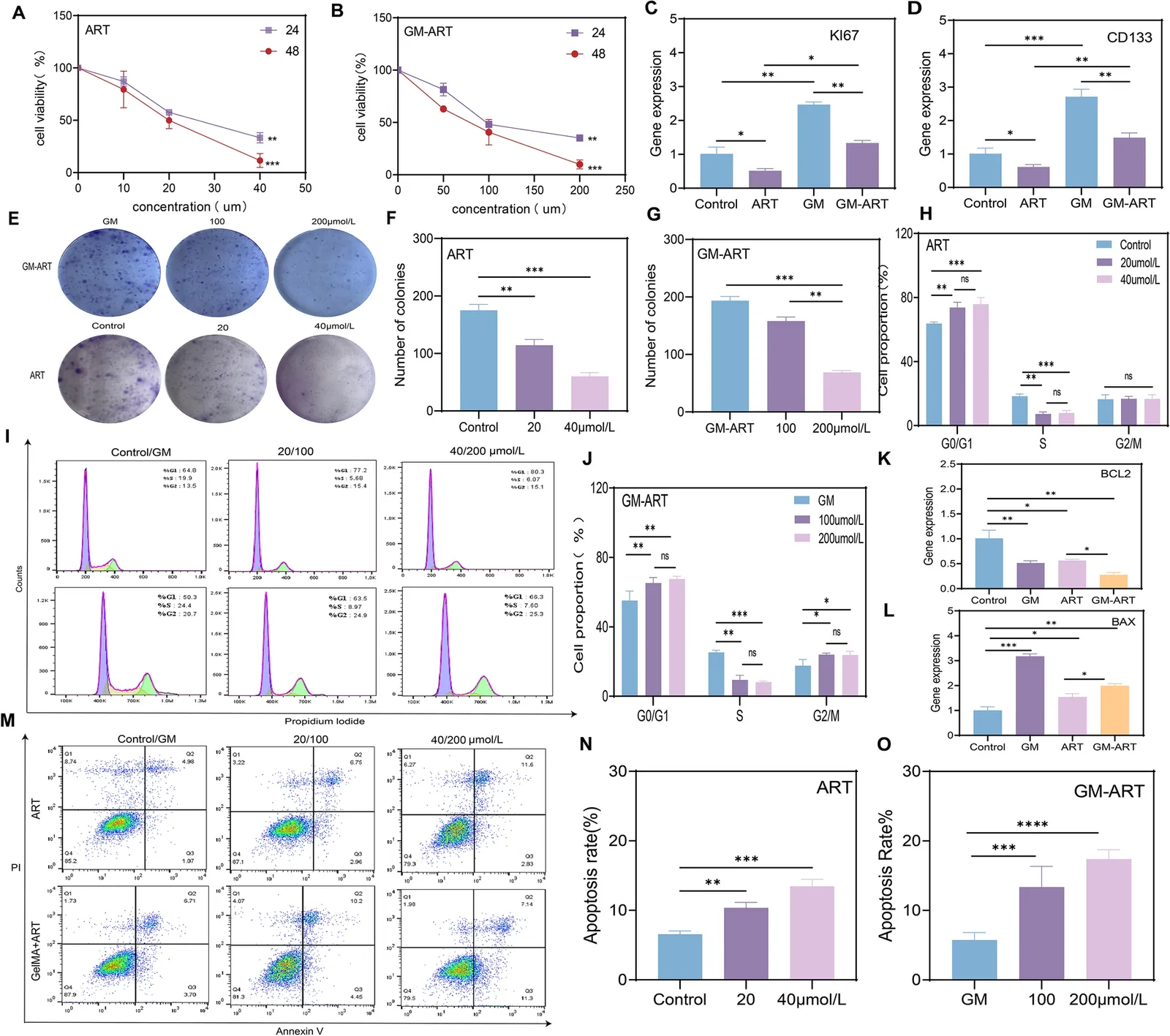
Effects of artesunate on proliferation, migration and cell cycle of ovarian cancer cells under different culture conditions. **A, B** Cell proliferation assays of artesunate-treated ovarian cancer cells under two-dimensional and three-dimensional GelMA microsphere culture conditions. **C, D** Expression of proliferation-related genes CD133 and Ki67 in 2D and 3D cultures. **E** Colony formation assays in 2D and 3D cultures. **F, G** Quantitative statistics of cloning experiments. **I** Cell cycle analysis of artesunate-treated cells under 2D and 3D conditions. **H, J**. Quantitative analysis of cell cycle experiments. **K, L** Expression of apoptosis-related genes. **M** Apoptosis assay of cells cultured in 2D and 3D GelMA microspheres. **N** Quantitative analysis of apoptosis experiments. (* indicates P < 0.05, ** indicates P < 0.01, *** indicates P < 0.001, ns indicates not significant)

To evaluate the proliferative response of ovarian cancer cells following ART treatment, equal numbers of cells were seeded into conventional 2D 24-well culture plates and 3D GelMA microsphere-based scaffolds, respectively. Once the cells reached approximately 70% confluence, they were treated with varying concentrations of ART. Cell viability was measured using the CCK-8 assay. The results showed that the concentration of ART required for 3D GelMA microsphere cultures was significantly higher than that for the 2D culture system, indicating that cells under 3D culture conditions exhibited stronger drug resistance (Fig. 3A, B). To further validate these findings, qRT-PCR was performed to assess the expression of cell proliferation-related genes, such as CD133 and KI67, indirectly reflecting the enhanced proliferative capacity of cells under 3D culture conditions. Compared to 2D cultures, ES-2 cells cultured in 3D GelMA microspheres showed a more pronounced upregulation of these genes. After ART treatment, cells cultured under 3D GelMA microsphere conditions also exhibited significant gene upregulation (Fig. 3C, D). This indicates that ovarian cancer cells proliferate significantly more on GelMA microspheres than in two-dimensional culture, and that gene downregulation following ART treatment of ovarian cancer cells is also significantly greater than in two-dimensional culture, demonstrating that three-dimensional culture models are more suitable for the screening of anticancer drugs. The colony formation assay results corroborated the CCK-8 assay findings, further confirming the impact of 3D culture on cell proliferation and drug resistance (Fig. 3E, F, G). Furthermore, the impact of ART on the cell cycle of ES-2 ovarian cancer cells was explored. The results indicated that under both 2D and 3D GelMA microsphere culture conditions, ART treatment caused cell cycle arrest at the G0/G1 phase. However, compared to 2D cultures, the percentage of cells in the G0/G1 phase was significantly lower in 3D GelMA microsphere cultures, while the percentages of cells in the S and G2/M phases were increased. These data suggest that 3D GelMA microsphere culture promotes DNA and histone synthesis in ES-2 cells, inducing a shift from the G1 phase to the S phase (Fig. 3H, I, J). Compared to 2D culture, cells cultured in 3D GelMA microspheres are generally less sensitive to chemotherapy drugs (Lai et al. 2011). Apoptosis is a complex process regulated by various pro- and anti-apoptotic molecules. The balance between anti-apoptotic and pro-apoptotic molecules determines the final fate of the cell. *BCL-2* and *BAX* are key regulators of apoptosis, with *BCL-2* enhancing the cell’s resistance to DNA damage and chemotherapy-induced apoptosis, while *BAX* forms homodimers to inhibit *BCL-2* anti-apoptotic function and promote apoptosis Newton et al. (2024). In ES-2 cells cultured in 3D GelMA microspheres, the mRNA expression of *BAX* was 2.38 times higher in 3D microspheres than in 2D culture, and after ART treatment, the *BAX* mRNA expression in 3D microspheres remained 1.33 times higher than in 2D culture. In contrast, the mRNA expression of *BCL‑2* in 2D culture was 1.98 times higher than that in 3D microspheres; after ART treatment, the *BCL‑2* mRNA expression in 2D culture was still 1.94 times higher than that in 3D microspheres (Fig. 3K, L)**.**To further explore the potential of 3D GelMA microsphere cultures in drug screening and their impact on cellular biological characteristics, this study used Annexin V-FITC double staining to assess the sensitivity of ovarian cancer cells to Artemisinin in both culture methods, with Annexin V serving as a specific marker for early apoptosis. In this experiment, ART was administered at concentrations of 20 μmol/L and 40 μmol/L in 2D culture, while cells in 3D GelMA microspheres were treated with 100 μmol/L and 200 μmol/L ART for 24 h. Subsequently, apoptosis was assessed using flow cytometry. The results showed that, although the apoptotic cell numbers were similar under both culture conditions following ART treatment, the cells cultured in 3D GelMA microspheres exhibited a stronger anti-apoptotic ability. Specifically, under the 3D culture system, tumor cells showed significantly enhanced drug resistance to Artemisinin, indicating that the 3D culture model more accurately reflects the drug resistance of tumor cells (Fig. 3M, O). This suggests that 2D culture may lead to false-positive results in drug screening, whereas the 3D GelMA microsphere culture system significantly improves the accuracy and efficacy of drug screening. This finding is crucial for tumor precision therapy and drug development, emphasizing the need to prioritize 3D culture models in drug screening to better simulate in vivo microenvironments and cellular responses.

### Evaluation of the biological effects of ART on ovarian cancer cells cultured on GelMA microspheres and in two‑dimensional cultures

**Fig. 4:**
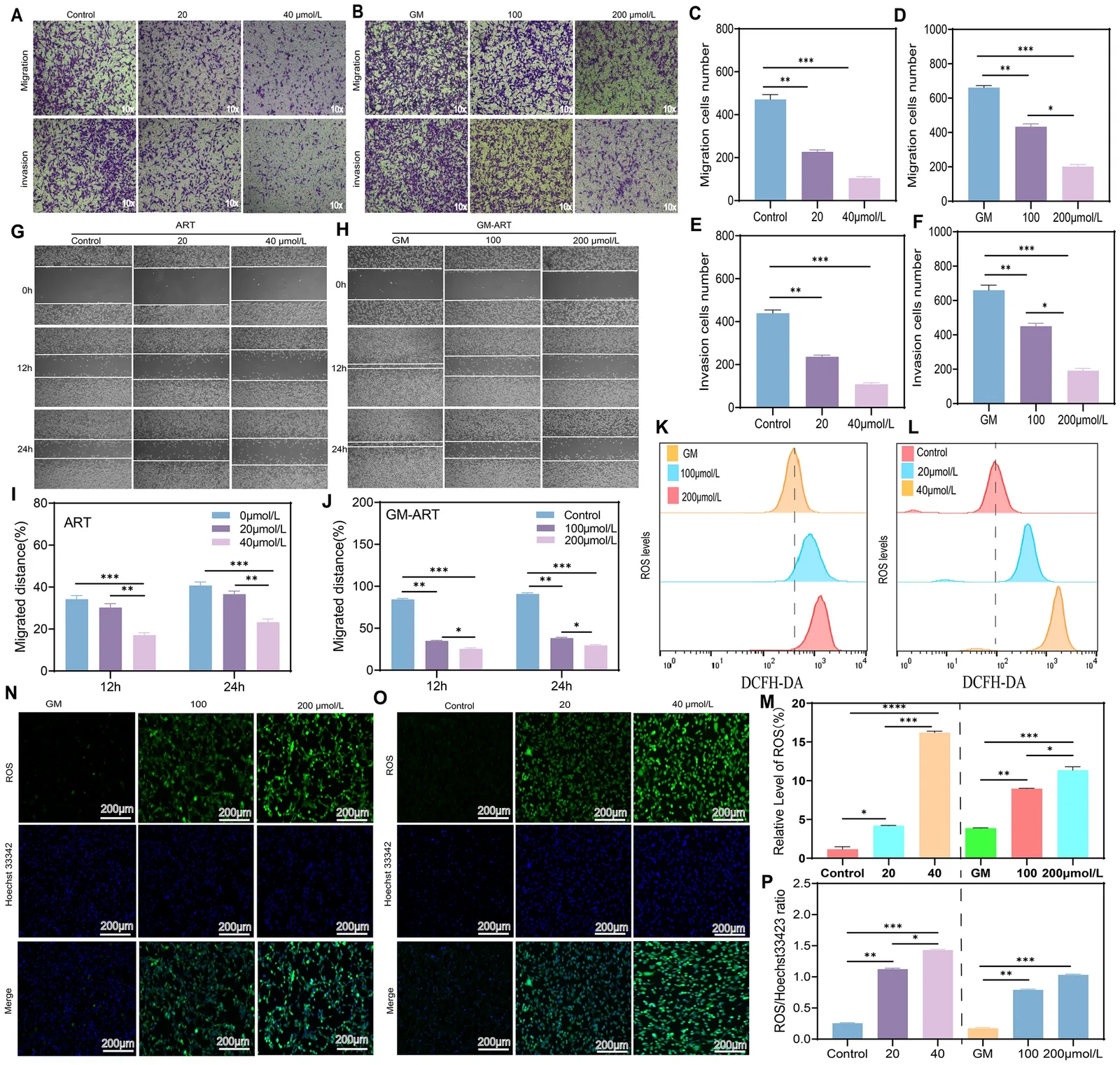
Effect of artesunate on cell migration, invasion and ROS in GelMA microspheres. **A, B** Cell migration and invasion assays under two-dimensional and three-dimensional GelMA microsphere culture conditions. **C, D, E, F** Quantitative analysis of cell migration and invasion. **G, H** Wound healing assays after artesunate treatment under 2D and 3D culture conditions. **I, J** Quantitative Analysis of Cell Migration and Invasion. **K, L** Flow cytometric quantification of ROS staining of GelMA microspheres and two-dimensional cultured cells. **M** ROS streaming detection quantitative statistical results. **N, O** ROS fluorescence staining of GelMA microspheres and two-dimensional cultured cells. **P** Statistical quantification of ROS staining of both cultures. (* indicates P < 0.05, ** indicates P < 0.01, *** indicates P < 0.001)

Tumor cell migration and invasion are critical steps in the metastatic process, directly influencing tumor growth, spread, and prognosis (Lichtenberg et al. 2023). Tumor cells employ a series of complex mechanisms to achieve migration and invasion, including alterations in cell–cell junctions, extracellular matrix (ECM) remodeling, dynamic reorganization of the cytoskeleton, and interactions with the surrounding microenvironment. During migration and invasion, tumor cells activate multiple signaling pathways, such as the Rho/ROCK pathway, PI3K/Akt pathway, and MAPK pathway, all of which regulate processes like cytoskeletal remodeling, cellular mechanical tension, cell–cell adhesion, and ECM degradation to promote cell migration and invasion (Noubissi Nzeteu et al. 2022**).** developed a novel 3D bladder cancer cell culture model using GelMA as a scaffold. Bladder cancer cells were cultured in both 2D and 3D environments and treated with rapamycin and Bacillus Calmette-Guerin (BCG) drugs. The results showed that the 3D model exhibited faster proliferation, higher stability, and greater drug resistance compared to the 2D model. Additionally, cancer cells cultured in 3D displayed lower drug sensitivity and simulated intercellular interactions and basal activities, closely resembling the in vivo environment (Kim et al. 2019; Loessner et al. 2016). In this study, ES-2 ovarian cancer cells were cultured in both 2D and 3D GelMA microsphere conditions. After 24 h of culture in the upper chamber of a Transwell, the migrated cells were stained with 0.1% crystal violet for 5 min and observed under a microscope. The results showed that, compared to the 2D culture, 3D GelMA microsphere culture significantly enhanced the migration of ES-2 ovarian cancer cells. Even after treatment with Artemisinin (ART), migration of cells cultured in 2D was slower, whereas the cells cultured in 3D GelMA microspheres exhibited faster migration. This indicates that 3D culture conditions can effectively enhance tumor cell migration ability, which is maintained even after drug treatment, consistent with the findings from the scratch assay. Additionally, under microsphere culture conditions, ES-2 cell invasiveness was significantly increased, Following treatment with ART, the invasive capacity of cells in 3D microspheres was significantly reduced, indicating that the drug inhibits migration and invasion of ovarian cancer cells even under 3D drug-resistant conditions. These results suggest that although 3D GelMA microspheres promote tumor cell migration and enhance invasiveness, these effects are markedly suppressed after ART treatment, further highlighting the value of ART as an anticancer agent in drug screening using GelMA microsphere-based models. These findings provide essential experimental evidence for further research into tumor cell biological characteristics and drug resistance mechanisms under different culture conditions. (Fig. 4A, B, C, D, E, F). In 3D cell cultures, cell migration is restricted by the three-dimensional spatial environment and primarily relies on microtubule dynamics, whereas in 2D cultures, cell migration is driven by myosin-mediated contraction, integrin-mediated adhesion, and actin filament-driven protrusions. Yamada and Sixt (2019). To investigate the effects of ART on the migration capacity of ovarian cancer cells under both 2D and 3D GelMA microsphere cultures, we performed a scratch assay to simulate the "wound healing" process and assess the migration rate. The results showed that under 3D GelMA microsphere culture conditions, the migration ability of ES-2 ovarian cancer cells was significantly higher compared to 2D culture conditions. However, even under the condition where the concentration of artesunate used in the 3D GelMA microspheres was five times that used in the 2D culture, the migration ability of ovarian cancer cells in the 3D culture system still showed a significant decrease, indicating that ART can effectively inhibit cell migration in the 3D microsphere model, which more closely mimics the in vivo environment (Fig. 4G, H, I, J).

Studies have shown that ART can induce excessive ROS production, leading to mitochondrial dysfunction and subsequently triggering cell apoptosis. In colorectal cancer cells, ART treatment significantly increases mitochondrial ROS production, leading to cell cycle arrest at the G0/G1 phase, and inhibits cell proliferation through p16 and p21-mediated cellular senescence (Xue et al. 2019). Ovarian cancer cells cultured on the surface of GelMA microspheres and in two-dimensional culture plates were harvested separately, co-cultured in confocal dishes, treated with artemisinin, and then analysed for intracellular ROS levels by flow cytometry (Fig. 4 K, L, M) and for ROS signals under a fluorescence microscope (Fig. 4N,O, P). The results showed that, regardless of whether assessed by flow cytometry or fluorescence microscopy, ROS levels in two-dimensional cultured cells were significantly higher than those in microsphere-cultured cells, indicating that artesunate (ART) treatment under two-dimensional culture conditions induced higher cell mortality and more significant ROS accumulation. However, the results also indicated that although the ROS levels induced by ART treatment in GelMA microsphere-cultured cells were lower than those in two-dimensional cultures, ART was still effective in inhibiting cell proliferation and promoting cell death.

Based on above, 3D GelMA microsphere culture not only more accurately reflects the biological characteristics of tumor cells but also demonstrates significant differences compared to traditional 2D culture systems in terms of cell proliferation, migration, and drug resistance. These results provide new insights and experimental platforms for drug screening and the study of tumor pathogenesis.

### RNA-seq analysis of ovarian cancer cells cultured on 3D GelMA microspheres

**Fig. 5:**
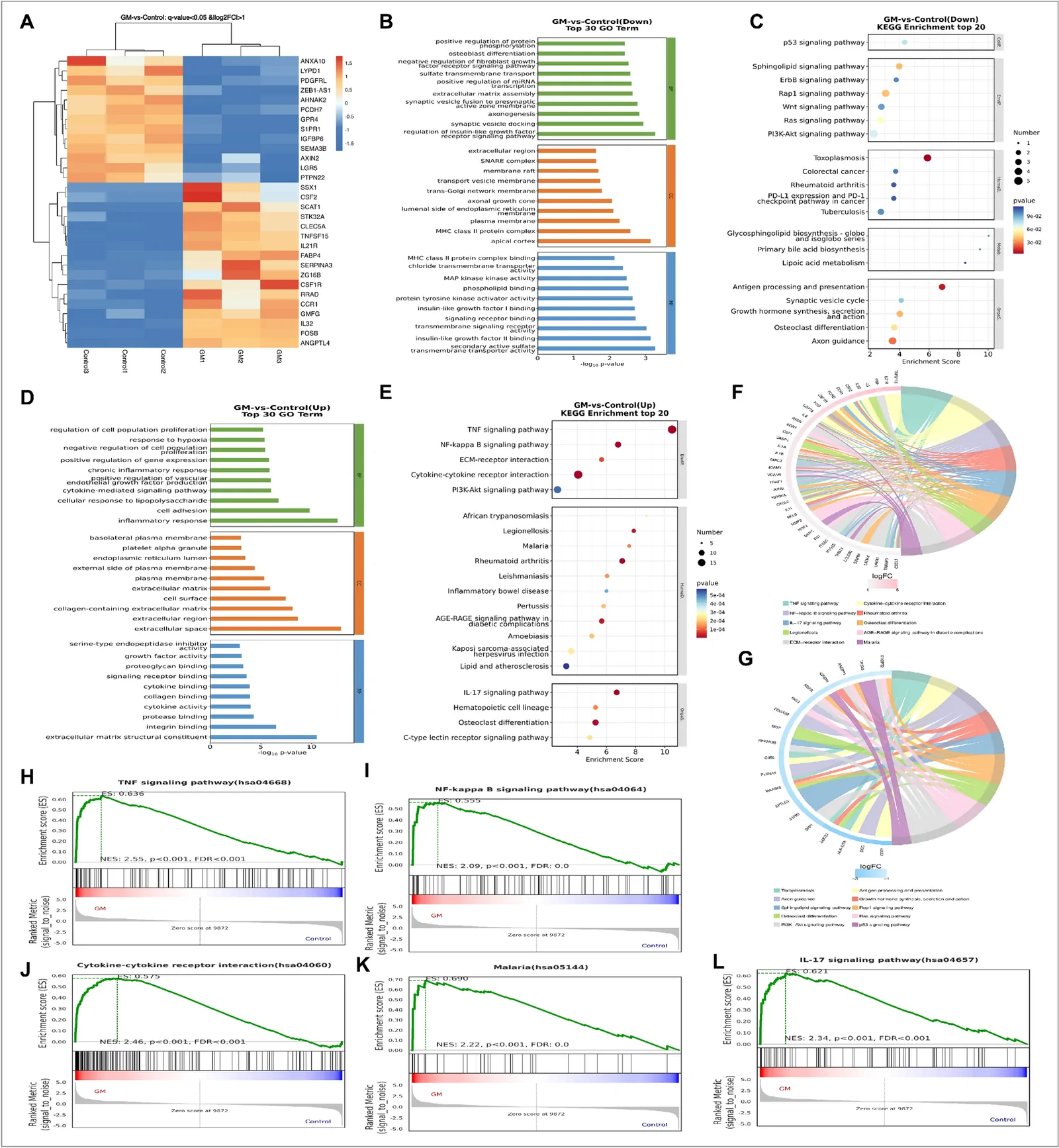
Two and three dimensional indication of RNA sequence analysis in culture. **A** 2D and 3D cluster analysis plots. **B**, **D** up- and down-regulated GO enrichment analysis between the two groups. **C**, **E** up- and down-regulated KEGG pathway enrichment analysis** .F**, **G** analysis of up- and down-regulated gene interactions involved in signaling pathways between the groups. **H**, **I**, **J**, **K**, **L** Enrichment analysis for GSEA.

To compare the overall transcriptomic profiles of ES-2 ovarian cancer cells cultured under two-dimensional and three-dimensional GelMA microsphere conditions, RNA sequencing was performed on the collected cells. Functional analysis using the Kyoto Encyclopedia of Genes and Genomes (KEGG) database revealed significant findings. Clustering analysis, presented as a heatmap, indicated that cells cultured in 3D GelMA microspheres enriched for genes associated with tumor proliferation, apoptosis, and invasion (Fig. 5A). For instance, *ANXN10、PDGFRL、SEMA3B、AXIN2、LGR5、FABP4、STK32A、CSF1R、FOSB、ANGPTL4* and so on. among these, Fatty Acid Binding Protein 4 (*FABP4*), a novel adipokine previously implicated in osteoarthritis (Zhang et al. 2024), has been shown to promote metastasis and confer cisplatin resistance in ovarian cancer cells induced by adipocytes (Mukherjee et al. 2020). In this study, we observed upregulated FABP4 expression in ovarian cancer cells cultured in 3D GelMA microspheres; this culture system may mimic the FABP4-mediated metastasis and drug resistance phenotypes observed in in vivo ovarian cancer tissues. However, this biomarker alone cannot fully demonstrate the functional similarity between the model and in vivo tissues; subsequent studies will employ techniques such as RNA sequencing to conduct systematic validation. Further Gene Ontology (GO) term enrichment analysis was conducted on the differentially expressed genes (DEGs). The resulting gene enrichment entries were categorized into three major categories: biological processes, cellular components, and molecular functions. GO analysis revealed that, compared to 2D culture, downregulated DEGs in the 3D culture were primarily enriched in the positive regulation of protein phosphorylation, negative regulation of fibroblast growth factor receptor signaling, positive regulation of miRNA transcription, and extracellular matrix (ECM) assembly (Fig. 5B). Conversely, upregulated DEGs were primarily enriched in the regulation of cell proliferation, hypoxia response, positive regulation of gene expression, chronic inflammation, positive regulation of vascular endothelial growth factor (VEGF) production, cytokine-mediated signaling pathways, cell adhesion, and inflammatory responses (Fig. 5D).KEGG pathway enrichment analysis further revealed that, relative to 2D culture, downregulated genes in the 3D culture were mainly enriched in the p53 signaling pathway, ErbB signaling pathway, Rap1 signaling pathway, Wnt signaling pathway, Ras signaling pathway, PI3K-Akt signaling pathway, and cancer-related *PD-L1* expression and *PD-1* checkpoint pathways (Fig. 5C). In contrast, upregulated genes were enriched in the TNF signaling pathway, NF-κB signaling pathway, ECM-receptor interaction, cytokine-cytokine receptor interaction, PI3K-Akt signaling pathway, and IL-17 signaling pathway (Fig. 5E).

The DEGs involved in these signaling pathways were further clustered. Notably, the downregulated pathways highlighted the *SEMA3B* protein as a particularly distinctive marker. *SEMA3* class signaling molecules are the only secreted proteins within the SEMA family and were originally identified as repulsive molecules for neural axon growth cones. SEMA3 has been shown to influence tumor regulation by either inhibiting or promoting cancer cell proliferation, metastasis, and angiogenesis. *SEMA3B* has been described as a potential tumor suppressor in lung cancer, esophageal cancer, and prostate cancer (Wang et al. 2020) (Fig. 5G). These results suggest that, in addition to the pro-angiogenic effects of Sema3E on tumor vascular endothelium, the Sema3E/Plexin-D1 signaling pathway may also promote cell invasion and migration in endometrioid ovarian cancer through the epithelial-mesenchymal transition (EMT) mechanism (Tseng et al. 2011). On the other hand, upregulated pathways pointed to CXC motif chemokine 3 (*CXCL3*), laminin α4 (*LAMA4*), thrombospondin-1 (*THBS-1*), prostaglandin-endoperoxide synthase 2 (*PTGS2*), integrin β5 (*ITGβ5*), fibronectin 1 (*FN1*), vascular cell adhesion molecule-1 (*VCAM-1*), interleukin-1β (*IL-1β*), caspase-1 (*CASP1*), early growth response 1 *(EGR1*), and growth differentiation factor 15 (*GDF15*) (Fig. 5F). Studies indicate that *CXCL3* promotes the proliferation and migration of cervical cancer, ovarian cancer (Jin et al. 2021 Jan), lung cancer, and oral squamous cell carcinoma cells through the MAPK/ERK signaling pathway (He et al. 2023). As key molecules of the extracellular matrix, laminins provide a delicate microenvironment for cellular function (Sun et al. 2017). *LAMA4* has been reported to play a role in endothelial formation and function, migration of inflammatory cells through the endothelium, and invasion of certain tumours. In addition, *LAMA4* is thought to play a role in cell migration, wound healing and angiogenesis (Thyboll et al. 2002). *LAMA4* has been identified as a key gene involved in the pathogenesis of ovarian cancer; overexpression of *LAMA4* significantly inhibits the proliferation, migration, and invasion of ovarian cancer cells. the upregulation of MEG3 increased the expression of *LAMA4* by sponging miR-30e-3p, which alleviated the malignancy of OC cells (Liu et al. 2020 May).Tumour angiogenesis is a hallmark of cancer (Hanahan and Weinberg 2011) and *VEGF* plays a central role in regulating tumour angiogenesis (Hoeben et al. 2004). *VEGF* has been shown to upregulate *VCAM-1* expression on endothelial cells (Kim et al. 2001). Growth differentiation factor 15 (*GDF15*) is a member of the transforming growth factor β (*TGF-β*) superfamily, also known as macrophage inhibitory cytokine 1 (*MIC-1*) (Eling et al. 2006), and elevated circulating levels of GDF15 have been clinically associated with cancer progression and chemotherapy resistance in ovarian, prostate, breast and colorectal cancers (Mimeault and Batra 2010; Paradiso et al. 2022; Roth et al. 2010).Moreover, Gene Set Enrichment Analysis (GSEA) results indicated that, compared to 2D culture, 3D culture showed significant positive enrichment in TNF signaling, NF-κB signaling, IL-17 signaling, cytokine-cytokine receptor interaction, and malaria gene sets. These results suggest that the change in culture conditions leads to substantial differences in gene set responses .(Fig. 5H, I, J, K, L).

### RNA-seq analysis of ovarian cancer cells treated with artesunate derivatives under different culture conditions

**Fig. 6:**
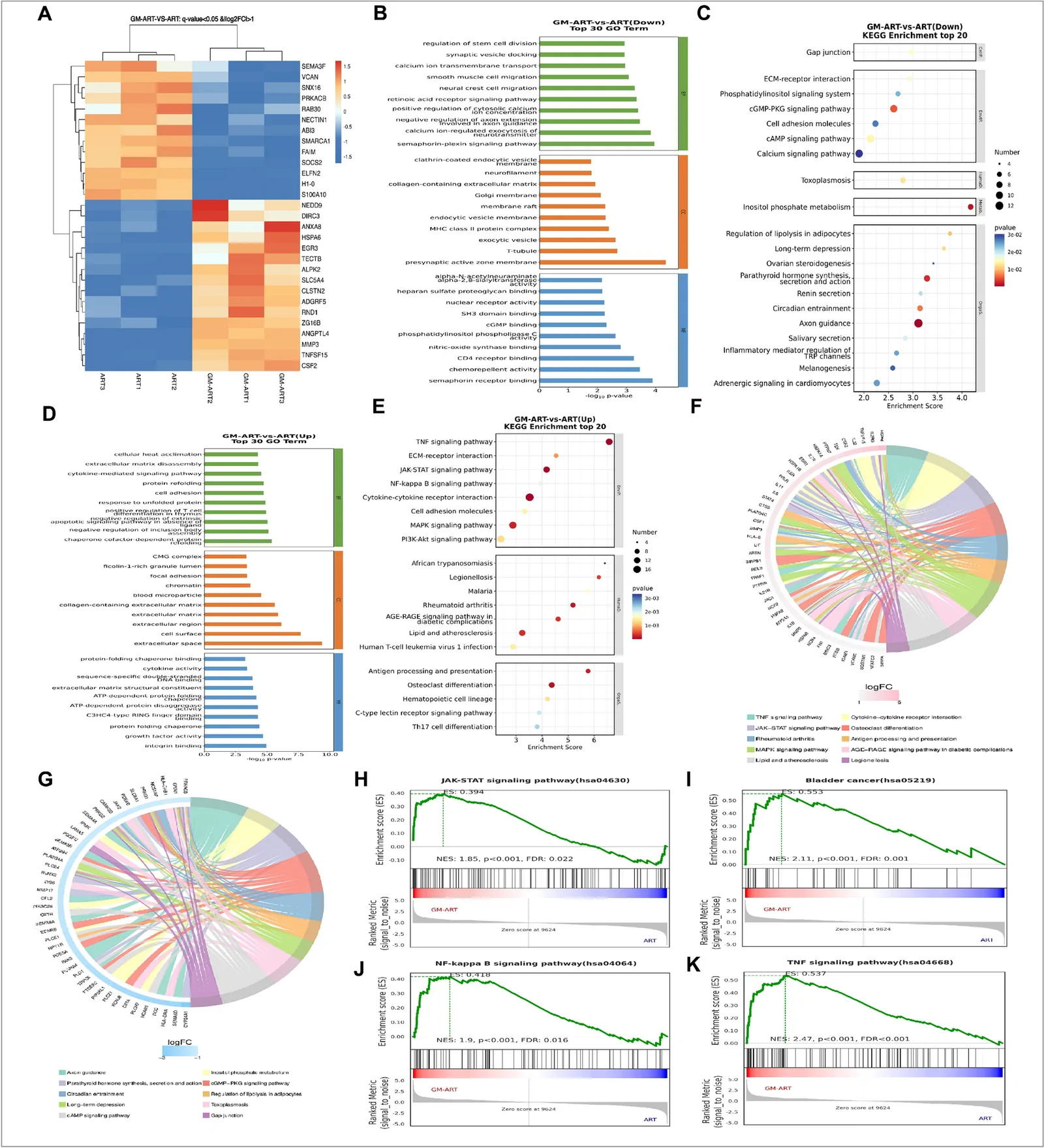
RNA sequence analysis after artesunate treatment in two and three dimensions indicating culture. **A** 2D and 3D cluster analysis plots.** B**, **D** up- and down-regulated GO enrichment analysis between the two groups. **C**, **E** up- and down-regulated KEGG pathway enrichment analysis. **F**, **G** analysis of up- and down-regulated gene interactions involved in signaling pathways between the groups. **H**, **I**, **J**, **K** for GSEA enrichment analysis.

To investigate the effects of artesunate derivatives on ovarian cancer cells in 2D and 3D GelMA microbead culture conditions, we performed RNA sequencing analysis on the treated cells. Cluster analysis and heatmap visualization revealed that, compared to 2D culture, 3D culture conditions It is enriched in genes associated with cell adhesion, such as *NEDD9, NECTIN1, ELFN2, CLSTN2 and ADGRF5. NEDD9* is a non-catalytic CAS family scaffolding protein that mediates multiple oncogenic factors and coordinates metastatic signaling. Its broad interactome regulates adhesion, migration, invasion, cell cycle, and survival. In most solid and hematologic malignancies, *NEDD9* promotes tumor growth, though it acts as a tumor suppressor in certain cancer types (Shagisultanova et al. 2015 Aug 1). Rashid et al. demonstrated that the expression of endogenous Nedd9 in tumor cells promotes the development and progression of ovarian cancer by broadly inducing oncogenic signaling and the expression of stem cell/mesenchymal genes (Gabbasov et al. 2018)(Fig. 6A).

In the subsequent Gene Ontology (GO) functional enrichment analysis of differentially expressed genes (DEGs), upregulated genes were predominantly enriched in extracellular matrix (ECM) degradation, cytokine-mediated signaling, protein refolding, cell adhesion, response to unfolded proteins, positive regulation of T-cell differentiation in the thymus, negative regulation of apoptosis signaling pathways, negative regulation of inclusion body assembly, and chaperone-dependent gene expression (Fig. 6B). Conversely, downregulated genes were primarily enriched in processes related to stem cell division regulation, smooth muscle cell migration, neuronal cell migration, retinoic acid receptor signaling, positive regulation of intracellular calcium ion concentration, negative regulation of axonal extension, axon guidance, calcium ion-mediated neurotransmitter exocytosis, and semaphoring -plexin signaling pathways (Fig. 6D). KEGG pathway enrichment analysis indicated that downregulated genes were mainly enriched in pathways related to gap junctions, ECM-receptor interactions, cGMP-PKG signaling, cell adhesion molecules, cAMP signaling, and ovarian steroidogenesis (Fig. 6C). On the other hand, upregulated genes were enriched in TNF signaling, ECM-receptor interactions, JAK-STAT signaling, NF-kappa B signaling, cytokine-cytokine receptor interactions, cell adhesion molecules, MAPK signaling, and PI3K-Akt signaling pathways (Fig. 6E).

Further enrichment analysis and chord diagrams suggested that several key markers were associated with downregulated pathways, including *RUNX2* (Runt-related transcription factor 2), *SEMA3B* (semaphorin 3B), and *PDGF-D* (platelet-derived growth factor D). *RUNX2* is a well-known regulator of osteoblast and chondrocyte differentiation, but emerging evidence also implicates its role in cancer progression and tumorigenesis, where its downregulation inhibits cancer cell proliferation (Song et al. 2023). In ovarian cancer, Tse et al. demonstrated that *SEMA3B* suppresses ovarian tumor formation in xenograft models (Tse et al. 2002). PDGF-D promotes tumor metastasis by increasing the expression of *CXCR4* and enhances renal cancer metastasis and angiogenesis through the upregulation of *MMP9* and *VEGF* expression (Liu et al. 2011; Xu et al. 2005). PDGF-D overexpression is an independent predictor of resistance to platinum-based chemotherapy; it may also serve as a potential biomarker for targeted therapy in epithelial ovarian cancer and is associated with a poor prognosis (Zhang et al. 2018). Moreover, PDGF-D plays a crucial role in epithelial-mesenchymal transition (EMT) (Fig. 6G) (Wu et al. 2013).For upregulated pathways, the analysis highlighted the key molecules *VLA4, NOX4*, and *HSPA8* (Fig. 6F). *VLA4* interacts with *VCAM1* on endothelial cells to mediate leukocyte trafficking during inflammation, and *VCAM1* activation leads to the phosphorylation of VE-cadherin, resulting in the loss of cell adhesion (Vockel and Vestweber 2013). In particular, levels of the inflammatory marker *VCAM*1 in blood, tumor tissue, and ascites samples serve as important predictors of surgical complexity and the risk of recurrence in advanced ovarian cancer (Gultekin et al. 2025). *NOX4* plays a pivotal role in chemotherapy and radiotherapy resistance in ovarian cancer cells. Its knockdown enhances the sensitivity to targeted therapy and radiotherapy by reducing the expression of HER3 and NF-κ B p65 (Liu et al. 2021). *HSPA8*, an important chaperone molecule, plays a critical role in protein folding monitoring and chaperone-mediated autophagy (CMA), which promotes the degradation of specific proteins (Ming et al. 2023) identified the critical role of the HSPA8/CAV1/β-catenin axis in the progression of treatment-resistant BRAF V600E colorectal cancer (CRC), highlighting the potential of *HSPA8* as a predictive biomarker and therapeutic target in clinical practice (Li et al. 2024b). demonstrated that caseinolytic protease P (*CLPP*) enhances cisplatin (DDP) resistance in ovarian cancer by inhibiting mitophagy and promoting cellular stress responses. Moreover, *HSPA8* facilitates the degradation of *CLPP* by regulating the stability of the *CLPP* protein (Kou et al. 2024).Moreover, the GSEA enrichment analysis under 3D culture conditions revealed that the JAK-STAT signaling pathway, bladder cancer, NF-kappa B signaling, and TNF signaling pathways were more significantly enriched in the 3D culture system, indicating that the genetic response to the drug treatment remains significantly different under 3D conditions (Fig. 6H, I, J, K).

### 3D GelMA microsphere-based culture promotes epithelial mesenchymal transition (EMT) in ovarian cancer cells

**Fig. 7:**
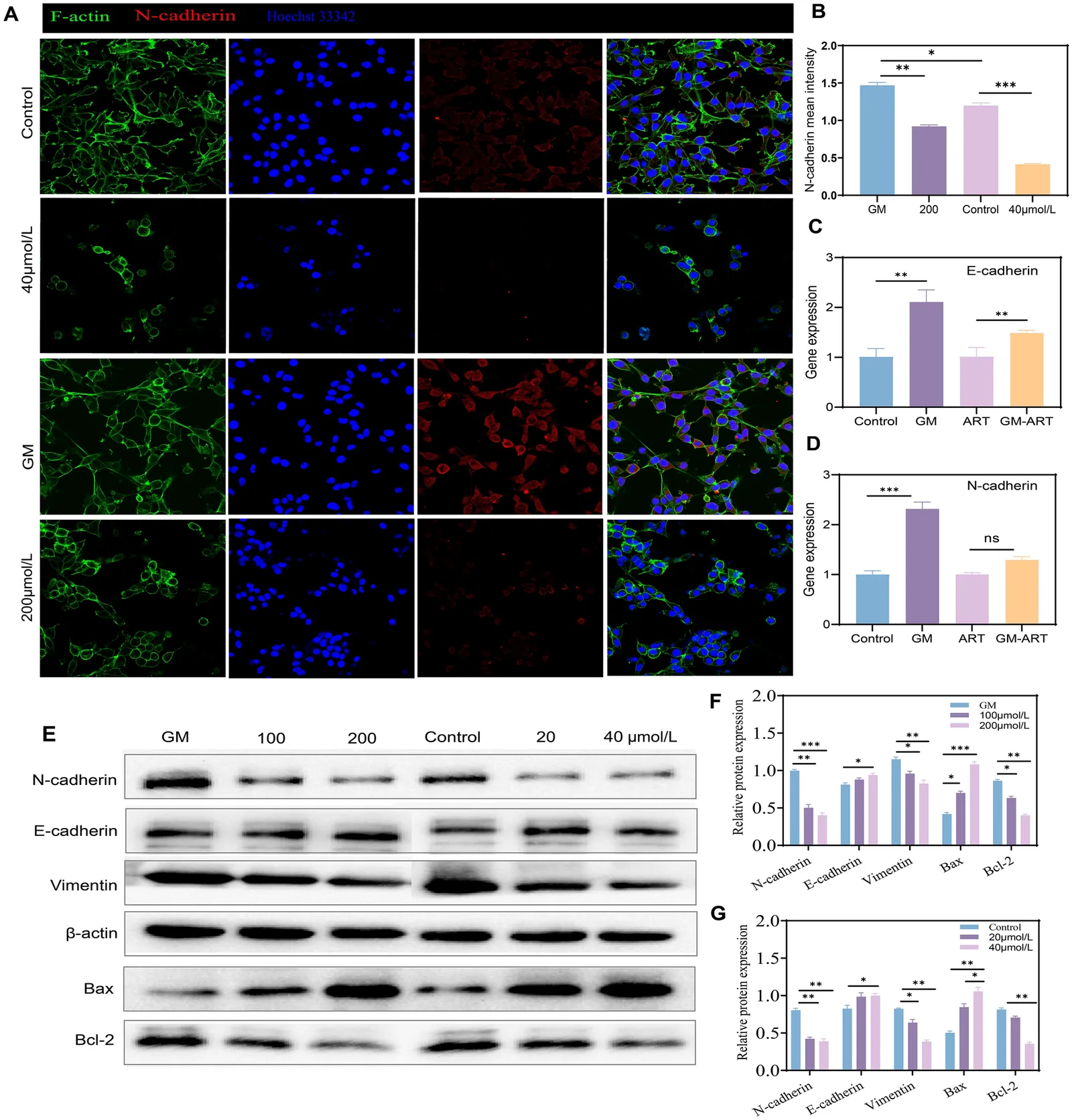
Effect of 2D and 3D culture on EMT in ovarian cancer cells. **A** Artesunate treatment in two and three dimensions indicating N-cadherin antibody staining after incubation. **B** the corresponding quantitative analysis of the meanfluorescence intensity (MFI) for N-cadherin. **C**, **D** *N-cadherin* and *E-cadherin* gene-related RNA expression. **E** EMT-related proteins (N-cadherin, Vimentin and E-cadherin) and apoptosis-related proteins (BAX and BCL-2) expression. **F**, **G** Western blot quantitative statistical analysis. (* indicates P < 0.05, ** indicates P < 0.01, *** indicates P < 0.001

Epithelial–mesenchymal transition (EMT) is implicated in various aspects of cancer malignancy, including tumor invasion, metastasis, and therapeutic resistance. During EMT, cancer cells progressively lose epithelial cell–cell adhesion and acquire a mesenchymal phenotype with enhanced migratory and invasive capacities (Luond et al. 2021). A hallmark of EMT is the dysregulation of cadherins, which destabilizes adherens junctions and triggers EMT-associated gene expression changes (Roy 2014). N-cadherin, a transmembrane glycoprotein mediating calcium-dependent intercellular adhesion, plays a pivotal regulatory role in cancer progression alongside E-cadherin (Barbaud et al. 2024). N-cadherin is predominantly expressed in the nervous system to mediate neuronal adhesion, while its expression in other normal tissues remains low. Recent studies have demonstrated that aberrant overexpression of N-cadherin enhances cancer cell motility and invasiveness, contributing to an aggressive tumor phenotype and facilitating tumorigenesis and metastasis (Sisto et al. 2021). Notably, downregulation of N-cadherin has been shown to reduce the invasiveness of esophageal squamous cell carcinoma and human melanoma cells (Liang et al. 2014; Li et al. 2009).In this study, ovarian cancer cells were cultured in both two-dimensional (2D) and three-dimensional (3D) GelMA microsphere systems, and N-cadherin expression was assessed via immunostaining (Fig. 7a). The results revealed that cells cultured in the 3D system exhibited significantly higher N-cadherin expression compared to those in 2D culture. Notably, even after artesunate (ART) treatment, N-cadherin expression remained elevated in the 3D-cultured cells. This finding was further confirmed by PCR analysis.

E-cadherin, an epithelial cell adhesion molecule, is essential for maintaining intercellular junctions. It localizes to the basolateral membrane by interacting with cytokeratins, forming adherens junctions that are critical for both adhesion and signal transduction (Shafraz et al. 2018). E-cadherin is widely recognized as a hallmark of epithelial cells and is considered a tumor suppressor. Loss or relocalization of E-cadherin from the membrane is indicative of EMT initiation (Fang and Kang 2021). During EMT, E-cadherin is downregulated or mislocalized, often due to the disassembly of the cytokeratin network, leading to a loss of cell polarity—a defining feature of epithelial differentiation and an early event in EMT (Ameen et al. 2001). E-cadherin-mediated adherens junctions connect to the cytoskeleton of neighboring cells, and remodeling of this structure is regulated through its cytoplasmic domain interacting with catenins, which, in turn, link to the actin cytoskeleton and regulate various tumor-related cellular processes (Cerdido et al. 2024).Vimentin, a type III intermediate filament protein, is another well-established marker of EMT and is typically upregulated during cancer metastasis (Wu et al. 2018). As a crucial component of the cytoskeleton alongside microfilaments and microtubules, Vimentin plays dynamic roles in structural support, adhesion, migration, and signal transduction (Danielsson et al. 2018). In the context of EMT and cancer progression, the vimentin filament network provides viscoelastic scaffolding that shields migrating cancer cells from mechanical stress and maintains organelle integrity, particularly of the nucleus (Patteson et al. 2019). It has been reported that Vimentin interacts directly with stress granules and invadopodia, mitigating endogenous stress caused by protein misfolding and enhancing cancer cell survival and invasion (Pattabiraman et al. 2020). In 3D cultures, Vimentin has been shown to be a prerequisite for generating protrusive forces, which are essential for confined-space migration, and its knockout leads to defective motility (Strouhalova et al. 2020).In our study, Western blot analysis revealed that in the EMT-related protein panel, N-cadherin and Vimentin were significantly upregulated, while E-cadherin expression was downregulated in 3D cultures, indicating a stronger EMT phenotype in ovarian cancer cells cultured within the GelMA microspheres. Additionally, the pro-apoptotic protein *BAX* was markedly elevated, whereas the anti-apoptotic protein *BCL-2* was reduced, suggesting enhanced apoptotic suppression in 3D cultures. Previous RNA sequencing results indicated that multiple genes associated with tumor cell invasiveness exhibited significant alterations under 3D culture conditions. Specifically, the expression levels of *SEMA3B, PCDH7,* and *VCAN* were markedly downregulated, whereas *ANGPTL4*, *ANXA8*, and *EGR3* were significantly upregulated in the 3D culture. These gene expression changes were further validated by comparing with normal ovarian epithelial cells (IOSE80) (Supplementary Fig. 1), confirming their close association with tumor cell invasiveness. Collectively, these findings suggest that ovarian cancer cells cultured on 3D GelMA microspheres display enhanced invasive potential and exhibit distinct biological behaviors compared to those cultured in 2D. This provides compelling evidence for the biological relevance of 3D culture systems and highlights their potential in advancing drug screening and optimizing therapeutic strategies.

## Conclusion

In summary, this study employed a three-dimensional (3D) GelMA hydrogel microsphere culture system to evaluate the effects of artesunate (ART) on ovarian cancer cells. Compared with conventional two-dimensional (2D) culture, the 3D system better mimicked the in vivo extracellular matrix (ECM) microenvironment, as evidenced by the increased drug tolerance of cancer cells and the requirement for higher ART concentrations to induce apoptosis. Transcriptomic analysis revealed significant upregulation of invasion-related genes and tumor-associated signaling pathways under 3D conditions. These findings highlight the advantages of 3D culture models in recapitulating the complex tumor microenvironment and improving the reliability of anticancer drug screening. Nevertheless, limitations remain, including the absence of vascular and immune components. Future studies should aim to develop more advanced multicellular co-culture systems and integrate high-throughput screening with multi-omics analysis to uncover novel therapeutic targets and synergistic drug effects. Collectively, this study not only demonstrates the potential of ART in ovarian cancer therapy but also underscores the broad applicability of GelMA-based 3D culture systems in cancer research.

## Materials and methods

### Materials and cells

The ovarian cancer cell line ES-2 and fetal bovine serum (FBS) were purchased from Wuhan Procell Life Science & Technology Co, Ltd. McCoy’s 5A medium was obtained from Shanghai Xiaopeng Biotechnology Co, Ltd. Agarose was purchased from Thermo Fisher Scientific. Gelatin was obtained from Beijing Solarbio. and methacrylic anhydride (MA) was purchased from Aladdin China. GelMA lysis buffer, droplet generation oil, and the photoinitiator were purchased from Chengdu Ruya Technology.

### The preparation of gelatin methacryloyl (GelMA)

Agarose powder (0.5 g) was dissolved in 50 mL of deionized water by heating and stirring at a temperature above 90 ℃ until fully melted, then stored at room temperature for later use.To synthesize GelMA, 40.0 g of gelatin was dissolved in 400 mL of PBS at 50 ℃ under constant stirring using a magnetic stirrer. Subsequently, 32 mL of methacrylic anhydride was slowly added, and the reaction was allowed to proceed. The reaction was then quenched by diluting the mixture fivefold with PBS. The resulting solution was dialyzed using a 10 kDa molecular weight cut-off dialysis membrane against deionized water for two weeks, with the water changed twice daily to remove unreacted methacrylic anhydride until the solution became clear. After dialysis, the solution was frozen at –80 ℃ overnight and then lyophilized using a freeze dryer for long-term storage.

### Hydrogel microsphere preparation

GelMA was dissolved in deionized water at 40 ℃ to a final concentration of 10% (w/v) by mixing 1 g of GelMA with 10 mL of deionized water. A photoinitiator (3%, v/v) was then added, and the solution was stirred thoroughly before being sterilized using a 0.22 μm filter. Monodisperse microspheres were generated using a droplet microfluidic device (EFL, China). The GelMA solution was injected at different flow rates using a micro-jet propulsion pump, where the dispersed phase formed uniform droplets under shear force. Droplet size was controlled by adjusting the flow rate difference between the continuous and dispersed phases. The collected droplets were crosslinked by exposure to 405 nm UV light for 1 min to form GelMA microspheres.The obtained microspheres were soaked in 75% ethanol overnight for sterilization, followed by washing with deionized water three times (20 min each) under sterile conditions. The microspheres were then freeze-dried to obtain porous GelMA hydrogel microspheres.Before seeding cells, an agarose layer (2 mL per well for 6-well plates) was applied to the bottom of each well to prevent adhesion. Approximately 15 mg of microspheres were required to cover the bottom of a 6-well plate, and about 3.2 mg for a 24-well plate.To collect the cells attached to the microspheres, the culture medium was replaced with a lysis buffer consisting of 1 mL complete medium supplemented with 6 μL of lysis reagent. After 4 h of incubation to allow GelMA degradation, the solution was centrifuged to collect the cell pellet.

### Characterization of microspheres

The microspheres were mounted onto SEM stubs using conductive adhesive tape and sputter-coated with gold for 45 s. Morphological characterization was performed using a scanning electron microscope (Apreo 2S, Thermo Fisher Scientific, USA) under an accelerating voltage of 10 kV. The average diameter and pore size of the microspheres were measured from randomly selected fields using Nano Measure 1.2 software.

### Biodegradability and biocompatibility of microspheres

To assess the degradation of the microspheres, 1 g of GelMA microspheres was added to 10 mL of serum-containing culture medium and incubated in a shaker at 37 ℃. The degradation status was evaluated after 10 days. For cell seeding, ES-2 ovarian cancer cells were inoculated onto the microspheres at a density of 1 × 10^6^ cells/mL. On day 5, samples were subjected to ethanol gradient dehydration and subsequently examined under SEM to observe the cell morphology on the microsphere surface.Furthermore, phalloidin staining and live/dead viability staining were performed on days 1, 3, and 5, respectively. Samples were visualized using an inverted fluorescence microscope and a laser scanning confocal microscope (Olympus, Japan).

### RNA sequencing Reverse Transcription, and qRT-PCR

Total RNA was extracted using TRIzol reagent (Vazyme, China) according to the manufacturer’s protocol. RNA purity and concentration were assessed using a NanoDrop2000 spectrophotometer (Thermo Scientific, USA), and RNA integrity was evaluated with an Agilent 2100 Bioanalyzer (Agilent Technologies, Santa Clara, CA, USA). RNA sequencing libraries were constructed using the VAHTS Universal V5 RNA-seq Library Prep Kit following the manufacturer’s instructions. Sequencing was performed on an Illumina NovaSeq 6000 platform, generating 150 bp paired-end reads. Raw reads in fastq format were processed using fastp software, removing low-quality reads to obtain clean reads for subsequent data analysis. Reference genome alignment was performed using HISAT2 software, and gene expression levels were quantified in FPKM. Gene read counts were obtained using HTSeq-count. Principal component analysis (PCA) and visualization of gene expression (counts) were performed using R (v3.2.0) to assess biological replicates. Differentially expressed genes (DEGs) were identified using DESeq2 software, with genes meeting the criteria of q-value < 0.05 and fold change > 2 or fold change < 0.5 defined as DEGs. Hierarchical clustering of DEGs was performed using R (v3.2.0) to visualize gene expression patterns across different groups and samples. A radar plot of the top 30 DEGs was generated using the R package ggradar to illustrate the expression changes of upregulated and downregulated genes. Subsequently, Gene Ontology (GO), KEGG Pathway, Reactome, and WikiPathways enrichment analyses were conducted based on a hypergeometric distribution algorithm to identify significantly enriched functional terms. Bar plots, chord diagrams, or enrichment circle plots of significant functional terms were visualized using R (v3.2.0). Gene set enrichment analysis (GSEA) was performed using predefined gene sets, with genes ranked by differential expression between the two sample groups. Enrichment of the predefined gene sets at the top or bottom of the ranked list was assessed. RNA sequencing and analysis were conducted by Shanghai Ouyi Biotechnology. (Shanghai, China).Total RNA was extracted according to the experimental requirements, and cDNA was synthesized using a reverse transcription kit (Vazyme, China). Quantitative real-time PCR (qRT-PCR) was performed using the SYBR Green Master Mix according to the manufacturer's instructions, with the synthesized cDNA serving as the template. The thermal cycling conditions were as follows: initial denaturation at 95 °C for 3 min, followed by 40 cycles of denaturation at 95 °C for 30 s, and annealing/extension at 60 °C for 30 s. The mRNA expression levels of the target genes (*BCL-2*, *BAX, Ki-67, CD133, E-cadherin, N-cadherin, ANGPTL4, ANXA8, EGR3, SEMA3B, PCDH7*, and *VCAN*) were quantified using the 2⁻ΔΔCt method to calculate the relative expression levels in each group.

### CCK-8 assay and colony formation assay

Log-phase ES-2 cells cultured in 2D and 3D GelMA microspheres were seeded at a density of 1 × 10^4^ cells/mL, 600 µL per well, in 24-well plates, with three replicates per group. The cells were incubated in a 37 ℃, 5% CO2 incubator for 24 h. When the cells were fully adherent (approximately 70% confluence), the control group was treated with McCoy’s 5A complete medium containing 10% fetal bovine serum. The experimental groups were treated with ART at final concentrations of 10, 20, and 40 μmol/L, while the 3D GelMA microsphere groups were treated with ART at concentrations of 50, 100, and 150 μmol/L for 24 and 48 h. CCK-8 (CCK-8; Kaiji Biotech, China) reagent (10 μL) was added to each well, and the cells were incubated for an additional hour. Absorbance at 450 nm was measured using a microplate reader (Aosheng Instruments, China), and cell proliferation curves were plotted. All experiments were performed in triplicate.

### Cell viability

Ovarian cancer cells (ES-2) were seeded onto GelMA microspheres, and live/dead staining was performed on days 1, 3 and 5 using 2 μM calcein-AM (for labelling live cells; Invitrogen, USA) and 4 μM propidium iodide (PI; for labelling dead cells; UElandy, China). Cell imaging was performed using a fluorescence microscope (Leica,Germany). The cell survival rate was quantified by calculating the ratio of Calcein-AM-positive cells to the total cell count (Calcein-AM and PI double-positive cells) using ImageJ software. Ovarian cancer cells cultured on GelMA microspheres or in two‑dimensional monolayers were treated with artesunate. Subsequently, live/dead staining was performed using the aforementioned method, and the results were statistically analyzed.

### Scratch assay

For the scratch assay, cells from routine two‑dimensional culture and those cultured on three‑dimensional GelMA microspheres were collected. After lysis and centrifugation, the cell pellet was resuspended and seeded into 6‑well plates. When the cell density reached an estimated 60–70%, two parallel scratches were made on the bottom of each well using a sterile 200 μL pipette tip. Floating cells were then removed by washing with sterile PBS or serum‑free medium. Images were captured under a microscope at 0 h. The medium was then aspirated, and complete medium was added. The cells were further incubated for 12 h and 24 h, and images were taken again at each time point. The migration rate was calculated as: Migration Rate (%) = (Initial Scratch Area – Migrated Area) / Initial Scratch Area × 100%.

### Transwell migration and invasion assays

ES-2 cells cultured in 2D and 3D GelMA microspheres were collected by centrifugation, washed with PBS or serum-free medium, and resuspended in serum-free medium.Migration Assay: The cells were resuspended to a density of 2 × 10^5^ cells/mL and 150 μL of cell suspension was added to the upper chamber(Corning, 0.4 μm,USA), while the lower chamber contained 600 μL complete medium with 15% FBS. After 24 h of incubation, the chambers were fixed with 4% paraformaldehyde for 20 min, stained with 0.1% crystal violet for 5 min, and washed three times with PBS. Migration was observed under a 100 × optical microscope. Invasion Assay: Matrigel was diluted with serum-free medium in a 1:4 ratio and placed in the upper chamber (40 μL) for 1.5–2 h. Cells were resuspended to a density of 3 × 10^5^ cells/mL and 150 μL was added to the upper chamber, while the lower chamber contained 600 μL complete medium with 15% FBS. After 24 h of incubation, the chambers were fixed and stained as in the migration assay. Images were taken under a 100 × optical microscope.

### Apoptosis assay

Cell apoptosis was assessed using an Annexin V-FITC apoptosis detection kit (Elabscience, China), and analyzed by flow cytometry (NovoCyte Advanteon, Agilent, USA). ES-2 cells cultured in 2D conditions or on 3D GelMA microspheres were seeded into 6-well plates at a density of 2 × 10^5^ cells per well. After complete adhesion, the cells were treated with complete medium containing various concentrations of artesunate (ART) for 48 h. Following treatment, the culture supernatants and cell pellets were collected by centrifugation. The cells were washed once with phosphate-buffered saline (PBS), centrifuged again, and resuspended in 500 μL of diluted 1 × Annexin V binding buffer. Subsequently, 5 μL of Annexin V-FITC reagent and 5 μL of PI reagent (50 μg/mL) were added to the cell suspension. The mixture was gently vortexed and incubated in the dark at room temperature for 15 min. After staining, the cells were diluted with PBS and analyzed by flow cytometry within 1 h. All experiments were conducted in triplicate.

### Cell Cycle Assay

Cell cycle distribution was analyzed using a cell cycle detection kit (Kaiji Biotech, China) and flow cytometry (NovoCyte Advanteon, Agilent, USA). ES-2 cells cultured under 2D conditions or on 3D GelMA microspheres were seeded into 6-well plates at a density of 2 × 10^5^ cells per well. After full attachment, the cells were treated with complete medium containing various concentrations of artesunate (ART) for 48 h. Following treatment, the cells were washed once with phosphate-buffered saline (PBS), collected by centrifugation, and resuspended in 1 mL PBS. The cell suspension was slowly added dropwise to 3 mL of pre-chilled 75% ethanol while gently vortexing. The cells were fixed overnight at 4 ℃. On the next day, the ethanol was removed by centrifugation. The cells were washed once with PBS, centrifuged again, and the pellet was resuspended in 500 μL RNase A solution and 450 μL propidium iodide (PI) solution (mixed at a ratio of 1:9). After incubation at room temperature for 30–60 min in the dark, the samples were subjected to flow cytometric analysis.

### ROS levels

Intracellular reactive oxygen species (ROS) levels were assessed using the DCFH-DA fluorescent probe. ES-2 cells cultured under 2D conditions or on 3D GelMA microspheres were seeded into 6-well plates at a density of 1 × 10^5^ cells/mL, 1 mL per well. After cell attachment, the cells were washed twice with phosphate-buffered saline (PBS). DCFH-DA was diluted in serum-free medium at a ratio of 1:1000 to a final concentration of 10 μmol/L( Solarbio, China). Then, 1 mL of the diluted DCFH-DA solution was added to each well, followed by incubation at 37 ℃ for 20 min in the dark. Subsequently, nuclei were stained by incubating with 5 μg/mL Hoechst 33,342 (Beyotime, China) at 37 ℃ in the dark for 30 min, followed by three washes with PBS.After incubation, the cells were washed with serum-free medium to remove excess dye, digested with trypsin, and collected for flow cytometric analysis. Representative fluorescence images were captured using a fluorescence microscope.

### Immunofluorescence staining

ES-2 cells cultured under 2D conditions or on 3D GelMA microspheres were fixed with 4% paraformaldehyde for 15 min at room temperature, followed by three washes with phosphate-buffered saline (PBS), each lasting 5 min. Cells were then permeabilized with 100 μL of permeabilization buffer for 10 min and washed three times with PBS (3 min per wash). Subsequently, the cells were blocked with 5% bovine serum albumin (BSA) for 1 h at room temperature. After removing the blocking solution, cells were incubated with a primary antibody against N-cadherin (1:200 dilution; 5 μL per 1 mL solution) for 2 h at room temperature. The cells were then washed with PBS three times (5 min each), followed by incubation with a secondary antibody (goat anti-rabbit IgG (H + L), 1:200 dilution, total volume 2 mL) for 1 h at 37 ℃. After three additional PBS washes (3 min each), the cells were stained with iFluor™488-phalloidin （1:200,Solarbio，China） at 37 ℃ for 1 h to visualize F-actin, followed by three PBS washes.Nuclei were counterstained with Hoechst 33,342 (10 μg/mL, Beyotime, China) for 10 min at room temperature and washed three times with PBS. Finally, samples were imaged using a laser scanning confocal microscope.

### Western blot analysis of protein expression

Primary antibodies against Bcl-2 (H651610022), Bax (H651381018), β-actin (H651614006), N-cadherin (H651465026), E-cadherin (ET1607-37), and Vimentin (ET1610-39) were purchased from HuaAn Biotechnology. (China). After 24 h of ART treatment, total proteins were extracted from ES-2 cells using RIPA lysis buffer. Protein concentrations were determined using the BCA protein assay. Equal amounts of protein were loaded and separated by SDS-PAGE, followed by transfer to PVDF membranes. Membranes were blocked with 5% non-fat milk for 1 h at room temperature and then incubated overnight at 4 °C with the corresponding primary antibodies. After five washes with PBST, membranes were incubated with HRP-conjugated goat anti-rabbit IgG (H + L) secondary antibody (Wuhan Sanying, China, Cat# SA00001-2). Protein bands were visualized using a chemiluminescence imaging system (Bio-Rad, China), and band intensities were quantified using ImageJ software for statistical analysis.

### Statistical Analysis

All data are presented as mean ± standard deviation (SD). Statistical analyses were performed using GraphPad Prism 8 software (GraphPad Software, USA). An unpaired two-tailed Student’s t-test was used for comparisons between two groups, and one-way analysis of variance (ANOVA) was applied for comparisons among multiple groups. A p-value < 0.05 was considered statistically significant.

## Supplementary Information

Below is the link to the electronic supplementary material.

Supplementary file1 (DOCX 106 KB)

## Data Availability

All data used to support the findings of this study are available from the corresponding author upon request.
