# Supplementary material for: The evaluation of biological effects of artesunate on ovarian cancer using three-dimensional cell culture methods
Source: Cell Tissue Bank. 2026 Aug 20;27(3):45. doi: 10.1007/s10561-026-10226-2 (PMC13493420; doi:10.1007/s10561-026-10226-2)
Supplement: Supplementary file 1 [file 10561_2026_10226_MOESM1_ESM.docx]

Supplementary Figure. 1
